# Improving Spatial Resolution of Multispectral Rock Outcrop Images Using RGB Data and Artificial Neural Networks

**DOI:** 10.3390/s20123559

**Published:** 2020-06-23

**Authors:** Ademir Marques Junior, Eniuce Menezes de Souza, Marianne Müller, Diego Brum, Daniel Capella Zanotta, Rafael Kenji Horota, Lucas Silveira Kupssinskü, Maurício Roberto Veronez, Luiz Gonzaga, Caroline Lessio Cazarin

**Affiliations:** 1 Vizlab|X-Reality and Geoinformatics Lab, Graduate Programme in Applied Computing, Unisinos University, São Leopoldo RS 93022-750, Brazil; marianne@edu.unisinos.br (M.M.); diebrum@unisinos.br (D.B.); dzanotta@edu.unisinos.br (D.C.Z.); khorota@edu.unisinos.br (R.K.H.); lkupssinsku@edu.unisinos.br (L.S.K.); VERONEZ@unisinos.br (M.R.V.); lgonzaga@unisinos.br (L.G.J.); 2 Department of Statistics, State University of Maringá, Maringá PR 87020-900, Brazil; emsouza@uem.br; 3 CENPES-PETROBRAS - Centro de Pesquisas Leopoldo Américo Miguez de Mello, Rio de Janeiro RJ 21941-598, Brazil; cazarin@petrobras.com.br

**Keywords:** Landsat, high resolution, prediction, multispectral, artificial neural network, Super-Resolution, classification, CAVE

## Abstract

Spectral information provided by multispectral and hyperspectral sensors has a great impact on remote sensing studies, easing the identification of carbonate outcrops that contribute to a better understanding of petroleum reservoirs. Sensors aboard satellites like Landsat series, which have data freely available usually lack the spatial resolution that suborbital sensors have. Many techniques have been developed to improve spatial resolution through data fusion. However, most of them have serious limitations regarding application and scale. Recently Super-Resolution (SR) convolution neural networks have been tested with encouraging results. However, they require large datasets, more time and computational power for training. To overcome these limitations, this work aims to increase the spatial resolution of multispectral bands from the Landsat satellite database using a modified artificial neural network that uses pixel kernels of a single spatial high-resolution RGB image from Google Earth as input. The methodology was validated with a common dataset of indoor images as well as a specific area of Landsat 8. Different downsized scale inputs were used for training where the validation used the ground truth of the original size images, obtaining comparable results to the recent works. With the method validated, we generated high spatial resolution spectral bands based on RGB images from Google Earth on a carbonated outcrop area, which were then properly classified according to the soil spectral responses making use of the advantage of a higher spatial resolution dataset.

## 1. Introduction

Remote sensing studies for geology, engineering, and agriculture are greatly improved by spectral information that makes it easier to identify materials, as well as biological and chemical properties through the spectroscopy [1,2,3,4].

Spectral information is usually given by the reflectance level for a specific wavelength that is emitted by a common source. The spectroscopy [1] study the reflected wavelengths of both visible and invisible light e.g., red, green, and blue colors, visible near-infrared (VNIR), short-wave infrared (SWIR), mid-wave infrared (MWIR), and long-wave infrared (LWIR) [4]. As different materials reflect the electromagnetic energy in different wavelengths, they can be identified by analyzing the reflectance, given a series of wavelengths that characterizes the spectral signature of the material [4,5].

This spectral signature can be helpful in the studies of mineral and petroleum geology, as it can be auxiliary to study of rocky outcrops and its compositions. These outcrops are inner layers of the earth that were exposed to the surface by erosion, by the Earth’s tectonic plates movement, or by human intervention.

The study of analogue outcrops is an important matter to the oil industry, due to the low resolution of seismic scale data in the study of actual reservoirs [6,7].

Considering the outcrop analogues is possible to differentiate bare soil from rock minerals, while other spectral studies also analyze the vegetation index, water pollution, soil humidity, and the presence of ore deposits for mineral exploration.

While these spectral signatures seems contiguous, most spectral sensors have limited spectral resolution grouping sets of wavelength responses into defined bands.

For example, multispectral imaging (MSI) sensors have tens of wide spectral bands, while hyperspectral imaging sensors (HSI) have hundreds of narrower bands, increasing the spectral resolution information [4].

Spectral images can be acquired through sub-orbital survey, which is the case of spectral sensors onboard of airplanes and unmanned aerial vehicles (UAV), and by orbital surveys, through orbiting satellites [1,2,3,4]. These techniques are part of the remote sensing studies that also include traditional imagery acquisition (photos), photogrammetry, Light Detection and Ranging (LiDAR) scannings, and radiometry.

Although the use of UAVs offers better spatial resolution due to its lower flight altitude and the possibility of integration of modern spectral sensors (HSI) [8], the use of satellite information can be considered a feasible solution due to the wide data availability and reduced cost for users. However, the spatial resolution of the orbital sensors is of the order of tens of meters for the free data of Sentinel, Aster, and Landsat satellites [9].

The satellites are grouped in constellations, where each constellation has its specific conditions. Common satellite data used for mineral exploration come from the Landsat and ASTER constellations, both managed by NASA projects [10]. The ASTER satellite has non-operational shortwave sensors since 2009 [11], giving LANDSAT more reliability in comparison to ASTER satellite, although the LANDSAT 7 has minor problems with the scan line corrector (SLC) since 2003 [12], but was successfully replaced by the LANDSAT 8 in 2013 [13].

The LANDSAT 8 satellite (https://www.usgs.gov/land-resources/nli/landsat/landsat-8) has a wide coverage and provides images with varying spatial resolution from 15m (panchromatic) to 100m (Thermal Infrared Sensor—TIRS) whereas other satellites with lower coverage or lack of spectral bands provide images with spatial resolution up to 0.31m (WorldView-4 satellite) [14].

The Google Earth services gather information from these satellites and provide free access under fair use, but still, a reduced number of bands are incorporated [15].

Due to the low spatial resolution of free satellite images provided by the USGS missions like Landsat and Aster (https://lpdaac.usgs.gov/data/get-started-data/collection-overview/missions/aster-overview/) compared to commercial satellite missions or suborbital remote sensing, a number of techniques have been created to solve this problem, where the most traditional method is pansharpening, i.e., data fusion of two images of the same area with different spatial resolutions with the aim of producing a single higher spatial resolution image [16]. The pansharpening technique has a variety of methods, where we can cite the Brovey transform, the wavelet fusion transform, the Gram–Schmidt transform and the IHS transform [14,17]. However, this method can present some limitations like the necessity of a panchromatic band, limitation in spatial resolution for this band, and systematic spectral distortion. These limitations inspired other techniques, based on supervised and unsupervised machine learning methods were also developed to increase spatial resolution [18,19,20].

Advanced techniques, like machine learning, are drawing attention into the geosciences. Several applications of artificial neural networks have proven useful for pattern recognition and for prediction of earth science events [21]. Recent works include the use of neural networks with the fusion of RGB images and sub-sampling of multispectral images [22,23]; the use of radial basis function networks to recover spectral information from RGB [24]; the RGB and hyperspectral unmixing based on matrix factorization [25]; and the use of sparse coding that is based on the works of [26] (in opposition to matrix factorization) and [22] (for spectral sparse recovery).

As an important part of machine learning techniques, the artificial neural networks (ANN) were designed in analogy to neurons and synapses. The artificial neurons (also called perceptrons) [27,28] send signals to others neurons through activation functions.

The learning of the network is done by calculating the error cost considering the actual and the desired output. This cost is then propagated (backpropagation) [27,28] in the network through optimizer function as the stochastic-gradient descent (SGD), ADAM or RMSProp [29]. Worth to mention variations of the ANNs, the Convolutional Neural Networks (CNNs) are generally used in signal and image recognition and generation, in one and two dimensions, respectively, through the use of filters or kernels (in convolutions) to seamlessly extract features from the image.

The advances in computational power and a great interest to resolve the high-resolution problems with Super-Resolution (SR) networks based on Convolutional Neural Networks (CNN) have arisen from the work of [30], which inspired works to improve the spatial resolution of spectral images [19] and image resolution in general [23,31,32,33,34,35], also influenced by the NTIRE [36] and PIRM2018 [37] contests. Most of them rely on benchmark datasets or specific datasets available only for the study. Furthermore, no one of them used or mentioned remote sensing data to this type of application. The reason might rely on the fact that most of these applications were developed to comply with visualization matters, not quantitative usage. However, with the even advanced of network architectures, the performance of spectral image super resolution is expected to be further improved and suitable to the remote sensing requirements. Moreover, to the best of our knowledge, the above mentioned approaches have not been tested to rock environments like outcrops and other kinds of minerals.

Although the application of CNN variations is a trend in generating high-resolution spectral information, they demand large datasets and are computationally expensive requiring high GPU and CPU processing power and multiple hours of training (30 h and up for 1000 epochs) as seen in [36]. Facing this scenario, this work aims to predict higher-resolution spectral images from a single RGB image using an artificial neural network that employs kernels from the image (like 2D convolutions) as input but without convolutional layers transformations.

Currently, through the use of classical images it is possible to determine erosion and land use chang [38,39]. Additionally, for ANN metrology, its possible to determine imprecise temporal-spatial parameters on images [40,41]. For this reason, one improving spatial solution on adverse resolution conditions are the implementation of Recurrent Neural Networks (RNN), Deep Reinforcement Learning (DRL), and Convolutional Neural Network (CNN) [42,43,44]. Consequently, this article shows that through images using RGB data and artificial neural networks, we improve the identification of carbonate outcrops for petroleum reservoirs.

As an application of this methodology, the CAVE dataset, composed with multispectral images of indoor scenes [45], was used alongside an extracted area from the Landsat 8 USGS database, as this area is of great importance to the geology studies of carbonated outcrops. This work extends a previous conference paper [46] by bringing a thorough validation of the proposed method downsizing the neural network input images to use the original size images as ground truth, by adding two spectral quality indexes to the two existing ones in the original paper, by including an additional dataset allowing us a proper comparison with previous work in the field, and by performing a supervised classification in both original and improved images.

## 2. Materials and Methods

This section describes the main method proposed, and the evaluation routines used for validation, after introducing the dataset used.

With the neural network models validated, the final products are assessed following a comparison image protocol and evaluation of common spectral indexes. As the final task, we performed soil classification in both the original and the increased spatial resolution images from Landsat in an outcrop area.

### 2.1. Proposed Method

The neural networks are organized in layers where each neuron in a layer receives the activation value of the previous layer adjusted by weight (fully connected sequential layers). This sum of multiplied weights and inputs is usually modified by an activation function e.g., sigmoid (0 to 1), Rectified Linear Unit (ReLu) (0 to inf), Tangent Hyperbolic Function (Tanh) (−1 to 1), and Softmax (0 to 1) [28]. An optimizer method is needed to update the weight values of the neurons given a cost or error function computed in the last layer (usually subtracting the desired value from the predicted value). The Adam optimizer [47] employed here uses momentum to avoid that the predicted values vary too much between updates (epochs) missing a possible optimum value.

The neural network architecture built ran under Python (version 3.7.6) language supported by the Anaconda package, with the integration of the Tensorflow library (version 2.1) for machine learning and the Keras library (version 2.31) abstraction layer for neural networks. Each training phase ran for 1000 epochs in a machine with a CPU Intel Core I5 7300HQ 2.5 GHz (3.5 GHz), 16 GB ram, and an Nvidia GTX 1050 (4 GB) GPU (ACER, São Paulo, Brazil).

**Figure 1:**
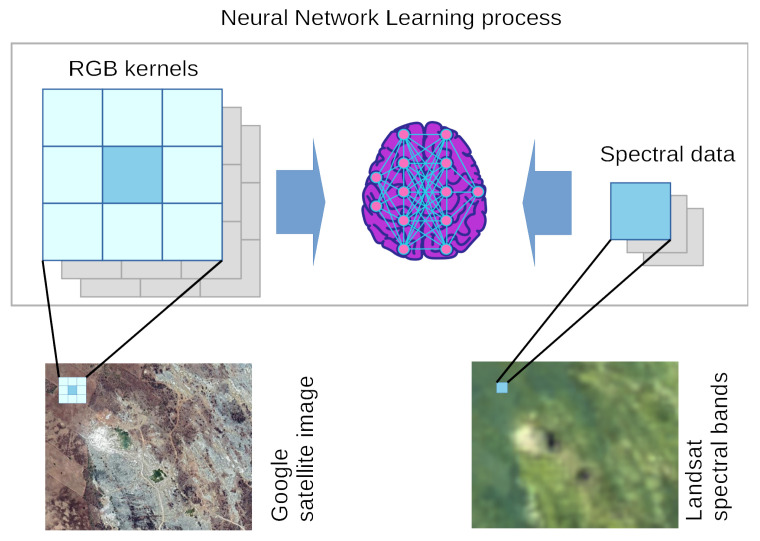
Kernel extraction and the neural network learning process. In this method, the RGB kernel (input) 3 × 3 × 3 is used to generate/predict 1 × 3 spectral bands (desired output).

The main difference between a pure sequential ANN and the structure built here, is that the input images are decomposed and all neighbors of a pixel are extracted considering its density in the image (how many bands there are). Given this, the network adjusts its input size to accommodate the proper neuron count given the training dataset, e.g., 3 × 3 neighbor pixels times 3 bands of the RGB image accounting for 27 input units. The Figure 1 illustrates this process.

For the output layer, the network was set to match the multispectral density of the desired output with 3 neurons for the Landsat 5, 6, and 7 bands, and 31 neurons for the CAVE dataset (see Section 2.4). For inner layers, the network was set to have 3 fully connected dense layers with 150, 70, and 35 neurons each.

The activation in the tree first layers used the Rectified Linear unit (Relu) activation function, while in the last layer the Sigmoid activation function was used to guarantee an output between 0 and 1. The input and output data were converted from and to 0–255 values standard in 8 bit image files.

### 2.2. Neural Network Validation

To validate the network models, the desired and predicted values in the test set are compared to let us know how good the predictive capacity of the model is. Common metrics include the mean squared error (MSE), mean absolute error (MAE) (that are also used as cost function during training), and the coefficient of determination $R^{2}$.

The MSE is given by

(1) $$MSE=\frac{1}{N}\sum_{i=1}^{N}{\left(y_{i}-{\hat{y}}_{i}\right)}^{2}$$

where ${\hat{y}}_{i}$ is the value predicted and $y_{i}$ is the expected value. This metric is more susceptible to outliers because the differences are squared, however a rooted MSE (RMSE) variation can be also used.

The MAE is given by

(2) $$MAE=\frac{1}{N}\sum_{i=1}^{N}|y_{i}-{\hat{y}}_{i}|$$

where ${\hat{y}}_{i}$ is the value predicted and $y_{i}$ is the expected value. In the MAE the differences have equal impact, being another way to estimate and evaluate the network and how its derivatives influences the model learning rate.

While the MSE and MAE show how models perform against each other, they do not explicitly tell how good the models are to predict the correct values. For this task, the coefficient of determination $R^{2}$ is employed.

The $R^{2}$ is given by

(3) $$R^{2}=1-\frac{\sum_{i=1}^{N}{\left(y_{i}-{\hat{y}}_{i}\right)}^{2}}{\sum_{i=1}^{N}{\left(y_{i}-{\bar{y}}_{i}\right)}^{2}}$$

where $\bar{y}$ is the mean of observed values of *y*. The resulting value will be in the range of 0 (or −∞) to 1, with 1 indicating a perfect fit between the predicted values and the expected values.

### 2.3. Spectral Quality and Comparison of Generated Products

As the primary objective of increasing the spatial resolution is to obtain finer multispectral images, the direct evaluation of these products should be carried in a proper way. According to the Wald’s protocol [48], a reference image with an equal resolution to the final product should be used for comparison, however, in the absence of such, the generated image must be degraded to the original resolution for direct comparison.

Following this protocol the indexes or coefficients of evaluation takes two images (or set of individual spectral bands), the first is the image with the original resolution (I) and the second is the higher resolution generated image (J), but resized to the same resolution as the original one, where each multi-spectral element (pixel) in the image is described by i. There are a number of quality indexes to evaluate improved spectral products, that are also used in works that employ data fusion pansharpening or knowledge-based spatial up-sampling, as the RMSE [16,22,49,50], ERGAS [19,20,49], SAM [19,25,31,32,51], UQI [14,19,20], and Q4 [16,19,20].

The first image index is the Root mean Square Root (RMSE) given by

(4) $$RMSE\left(I,J\right)=\sqrt{E{\left(I-J\right)}^{2}}$$

where the differences between each image *I* and *J* are squared and accumulated and then rooted been the most simple of the quality image indexes. The optimal value for the RMSE is 0, however, it does not consider the spectral and spatial differences between the images [49] and is sensitive to the data range [52]. The Erreur Relative Globae Adimensionnelle de Synthèse (ERGAS) tries to overcome the RMSE downfalls accounting the resolution differences. The ERGAS index is given by

(5) $$ERGAS=100\frac{h}{l}\sqrt{\frac{1}{K}\sum_{k=1}^{K}{\left(\frac{\mathrm{RMSE}(k)}{\mu (k)}\right)}^{2}}$$

where μ(k) is the mean of a band *k* and *K* is the number of bands.

The Spectral Angle Mapper (SAM) measures the angle dissimilarity between two sets of spectral bands where the optimal value is 0, indicating no distortion [53]. The SAM is given by

(6) $$SAM\left(I_{\left\{i\right\}},J_{\left\{i\right\}}\right)=arccos\left(\frac{\langle I_{\left\{i\right\}},J_{\left\{i\right\}}\rangle }{\left|\right|I_{\left\{i\right\}}\left||\;|\right|J_{\left\{i\right\}}\left|\right|}\right),$$

Another index created to overcome the limitation of RMSE is the Universal Quality Image Index (UQI) [16,54], given by

(7) $$Q\left(I,J\right)=\frac{\sigma_{IJ}}{\sigma_{I}\sigma_{J}}\frac{2\bar{IJ}}{{\left(\bar{I}\right)}^{2}+{\left(\bar{J}\right)}^{2}}\frac{2\sigma_{I}\sigma_{J}}{\left(\sigma_{I}^{2}+\sigma_{J}^{2}\right)}.$$

This index is a combination of three components, where the first is the linear correlation, the second is the luminance proximity, and the third is the contrast similarity. The ideal value for this index is 1 (if the images are equal) in a 0 to 1 range. A modification of this index is the Q4 that considers the distortion between 4 bands, thus not used in this work.

### 2.4. Datasets

To validate the network and generate the final products a synthetic dataset and a Landsat dataset where used. The final objective is to obtain a high-resolution outcrop image generated from Landsat and an RGB image to properly detect carbonate soil areas.

#### 2.4.1. CAVE Dataset

**Figure 2:**
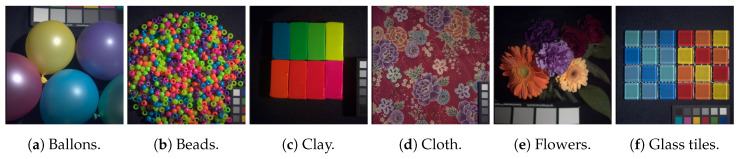
RGB representations of the multispectral sets from the CAVE dataset used in this work for validation.

The CAVE dataset [45] is a common [25,31,34,37,50] dataset for spectral reconstruction and/or Super-Resolution validation. It consists of 32 varied scenes grouped in “stuff”, “skin and hair”, “paints”, “food and drinks”, and “real and fake” images. The images have a spatial resolution of 512 × 512 pixels composed of 31 bands in the 400 mm to 700 mm wavelength range in intervals of 10 nm. The image sub-set chosen for this work is shown in Figure 2.

#### 2.4.2. Lajedo Soledade

**Figure 3:**
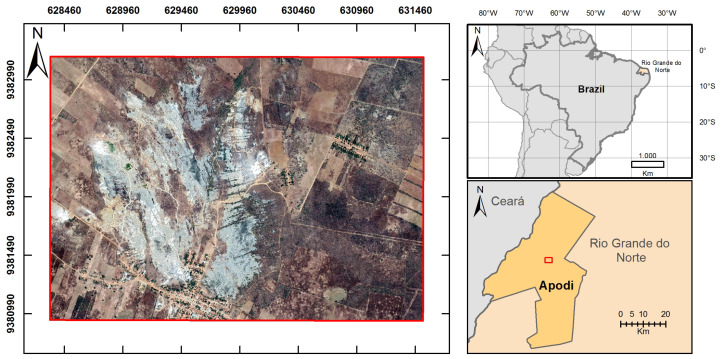
Study area, located at Soledade in the Apodi plateau as detailed in the map. Below the location figure is the image used for network training in WGS-84 reference system and UTM projection zone 24S.

The area of study is located at the Lajedo Soledade in the city of Apodi, state of Rio Grande do Norte, Brazil as detailed in Figure 3. The geology of the municipality consists of sedimentary rocks of the Potiguar Basin and the Aluvionares deposits [55,56,57]. The selected area used in this study belongs to the Jandaíra Formation in the Apodi plateau, whose composition is related to carbonaceous rocks with fossil molds gastropods and plants, deposited on sandstones of the Açu Formation.

The data acquisition was performed by searching the catalog of available images from the Landsat 8 satellite provided by the USGS. The satellite images were acquired using the Semi-Automatic Classification Plugin (SCP) [58] used in QGIS version 3.4.5, where it was also used to apply a traditional pansharpening technique to increase the spatial resolution of the spectral bands from 30 to 15 m per pixel, totalizing 213 × 151 pixels. These spectral images were originally acquired from the Landsat 8 in the time interval from 11/13/2018 to 11/27/2018, that have 2% of cloud cover. The higher resolution image (RGB) was provided by Google Earth from the Digital Globe constellation (time acquisition: 10/24/2018) and extracted with a resolution of 1m per pixel, totaling 3207 × 2278 pixels.

For training, the spectral bands 5, 6, and 7 of the Landsat 8 satellite were used with a spatial resolution of 30 m. Bands 5, 6, and 7 refer to the Near Infrared (NIR) (850 to 880 nm), SWIR1 (1570 to 1650 nm), and SWIR2 (2210 to 2290 nm) range. In addition, for training, we used the RGB image of Google Earth in the visible range (400 to 700 nm) degraded to a spatial resolution of 30 m.

The Landsat 8 images were preprocessed through SCP. The first preprocessing step was the conversion of Digital Numbers to Top of Atmosphere Reflectance (TOA), this conversion is needed because the digital numbers are not calibrated physical values, the conversion from DN to TOA allows to represent physically the reflectance contribution of elements as clouds, aerosols, and gases. The SCP uses the MTL files to extract the parameters needed to make the conversions. For the conversion of the digital numbers of the multispectral bands to TOA values, the reflectance rescaling coefficients are extracted from MTL files, if the user needs to work with thermal bands, the MTL files also provide the thermal constants (e.g., K1 and K2) for TOA brightness temperature conversion.

#### 2.4.3. Simulated Tests

To validate our methodology we used the CAVE dataset alongside a Landsat dataset given by the area presented prior. Respecting the Wald’s protocol and to compare with other works we downgraded the input images used for training, defining certain ratios between the original image using the spectral quality indexes present in Section 2.3.

These ratios scaled the original image by dividing its sides’ size by 2, 4, 8, 16, and 32, using nearest-neighbor interpolation. After using the downscaled RGB and multispectral images for training, the trained neural network received the original size RGB image to predict the multispectral image of equal size.

In the case of the Landsat image, a larger area covering the Lajedo Soledade was used for testing. In addition, the bands 4, 3, and 2 from Landsat were used instead for input. This was done to avoid the mosaic of multiple satellite sources, which can make it difficult for the neural network to learn, as for the larger area it was necessary in order to downsize the images and have enough training data.

After validating the neural network and the image products generated during the simulated tests, we proceed with the prediction of the high spatial multispectral image using the RGB image from Google, that was then evaluated following the same protocols presented in the Section 2.3, and used for spectral classification presented in the following section. The test dataset, studied area images, and code are available at [59].

### 2.5. Spectral Image Classification

Image classification techniques allow categorizing information using three different approaches. The first one is the Supervised Classification, which uses the knowledge of the expert to classify the image through the delimitation of Regions of Interest (ROI) that are used by the algorithms. The second approach is the Unsupervised Classification, which is based on clustering techniques that aim to partition the data into a given number of groups. The third approach is the Object-Based Classification, this one uses techniques to segment the images, differently of the pixel-based techniques, those create objects that represent the real land cover, however, the application of object-based classification requires larger amounts of computer memory.

In this work we chose the Supervised Classification approach because it allows to control how many classes will be created, besides that, commercial software like ENVI has various supervised algorithms, as the Maximum Likelihood, Neural Networks, Spectral Angle Mapper, Support Vector Machines, among others. Unsupervised methods were not selected because they do not allow the user to control how the classes are created, but only the number of partitions that the algorithm must divide the information. Object-Based methods were not selected due to the high computational requirements and also because of the different spatial resolutions of the images, which do not allow us to define the same objects in both images.

The classification process used in this work is divided into a series of steps as follows: The first step is the input of the Landsat 8 and the predicted images. The classification must be applied in both images in order to compare how good the spectral information predicted by ANN is to categorize the image information. Then we selected the algorithm used to classify both images; the second step, the Maximum Likelihood (ML) method [60] was chosen for the classification in ENVI 5.5 image processing software. In this algorithm, the pixels are classified according to the maximum likelihood between the classes by calculating the following discriminant functions for each pixel in the image:

$$g_{i}\left(x\right)=1np\left(\omega_{i}\right)-\frac{1}{2}1n\left|\sum i\right|-\frac{1}{2}{\left(x-m_{i}\right)}^{T}\sum i^{-1}\left(x-m_{i}\right)$$

where *i* is the class, *x* is the n-dimensional data (where *n* is the number of bands), $p(\omega_{i})$ is the probability that class $\omega_{i}$ occurs in the image and is assumed the same for all classes, $|\sum_{i}|$ is the determinant of the co-variance matrix of the data in class $\omega_{i}$, $|\sum_{i}^{-1}|$ is its inverse matrix, and $m_{i}$ is the mean vector.

After the selection of the algorithm, we set the parameters needed to perform the classification, which are divided into two main stages:

(a) Define the number of classes: In this first stage, we defined how many land cover categories exist in each image based on visual analysis. In this analysis, three classes of land cover were defined considering the two images:
  - Grassland: Composed by grass, undergrowth vegetation, and bushes;
  - Forest: Composed by dense vegetation;
  - Exposed Soil: Composed by rock outcrop, soil without cover of vegetation, urban areas, or water bodies.
(b) Collect the samples for each class: In this second stage of the parameter settings, the regions of interest were selected. Those regions consist in polygons that were collected considering each image, as Landsat 8 had a lower spatial resolution, we could not identify the geometric details with the same quality than in the predicted image. To solve this problem, we defined the regions of interest where we were certain about the respective class, collecting different ROIs for each image.

With the classification parameters set, the algorithm was applied for each image.

### 2.6. Classification Evaluation

As important as the classification is the validation process because it allows evaluating the performance of the classifier. This process is performed in ENVI using the ROIs provided by the user to classify the images.

From the selected ROIs it is possible to generate a random sample that can be considered the ground truth to evaluate the quality of the image classification process. The confusion matrix, which shows whether a certain image pixel was classified correctly or if the classifier assigned the value to another class, was built. From the confusion matrix were calculated some indices such as accuracy, Precision, Recall, kappa coefficient, Matthews Correlation Coefficient (MCC), for each of the classified images.

The accuracy shows how our model classified correctly the True Positives (TP) and True Negatives (TN) considering all the possible predictions to be made by the classifier (Equation (8)).

(8) $$Accuracy=\frac{TP+TN}{TP+TN+FP+FN}$$

where FP is the false positive class and FN is the false negative class. The accuracy can vary between 0 and 1, but it can be expressed as the hit percentage of the classifier.

The Precision shows how the classifier worked to classify positive values correctly, it is given by:

(9) $$Precision=\frac{TP}{TP+FP}$$

The precision given in the Equation (9) is very important to verify how the classifier assigns pixels of the second class to the class we want to predict. In other words, the higher the number of FP, the lower the precision measure.

The Recall is a similar measure to the Precision, but its importance consists of showing how our classifier can correctly predict the TP considering that some pixels can be assigned to the FN class:

(10) $$Recall=\frac{TP}{TP+FN}$$

The kappa coefficient [61] shows the degree of agreement between the classified pixels and the Ground Truth, it can be expressed by:

(11) $$k=\frac{N\times \sum_{i=1}^{n}m_{i},i\left(G_{i}C_{i}\right)}{N^{2}-\sum_{i=1}^{n}\left(G_{i}C_{i}\right)}$$

where *N* is the total number of classified values compared to truth values, *i* is the class number, $m_{i}$, *i* is the number of values in the main diagonal of the confusion matrix, $C_{i}$ is the number of predicted values belonging to the determined class, and $G_{i}$ is the number of truth values that belong to that class. This coefficient varies between 0 and 1, where 0 indicates no agreement between the predicted and truth values, while 1 indicates a perfect agreement.

The MCC [62] is important because it considers all possible prediction class, using the balance ratios of the four categories of the confusion matrix, as can be seen in Equation (12):

(12) $$MCC=\frac{\left(TP\times TN-FP\times FN\right)}{\sqrt{\left((TP+FP)(TP+FN)(TN+FP)(TN+FN)\right)}}$$

Different from the kappa index, MCC varies from −1 to 1, where −1 represents a completely wrong classification, while 1 represents a completely correct classification.

After the application of the Maximum Likelihood method to classify the Landsat 8 and the predicted images, the confusion matrices were generated and the validation metrics were calculated.

## 3. Results

This section presents the results for the predicted high spatial resolution multispectral images and the spectral classification comparison between the predicted high spatial resolution and the original NIR and SWIR bands from Landsat.

### 3.1. Improving Spectral Resolution

Following the proposed methodology to improve spatial resolution and to evaluate the generated products we present the results for multispectral images of the CAVE dataset and the Landsat satellite bands.

#### 3.1.1. CAVE Dataset

**Figure 4:**
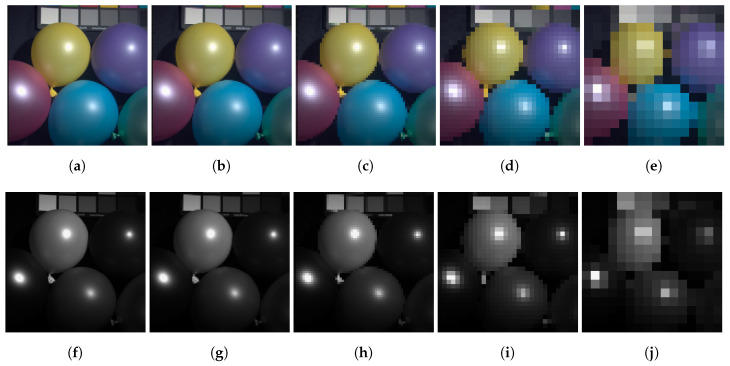
Balloon’s set example of the resized inputs used during training for each image in the CAVE dataset, for the ratios of 2 (**a**,**f**), 4 (**b**,**g**), 8 (**c**,**h**), 16 (**d**,**i**), and 32 (**e**,**j**), with resolutions of 256 × 256, 128 × 128, 64 × 64, 32 × 32, and 16 × 16 pixels.

The first results are from the validation of the methodology itself, firstly with the CAVE dataset and then with a Landsat test area. To illustrate and validate the model the input images for each training test were resized dividing its sides by 2, 4, 8, 16, and 32 as stated in the methodology section. Figure 4 illustrates this degradation for the Balloon multispectral set.

**Figure 5:**
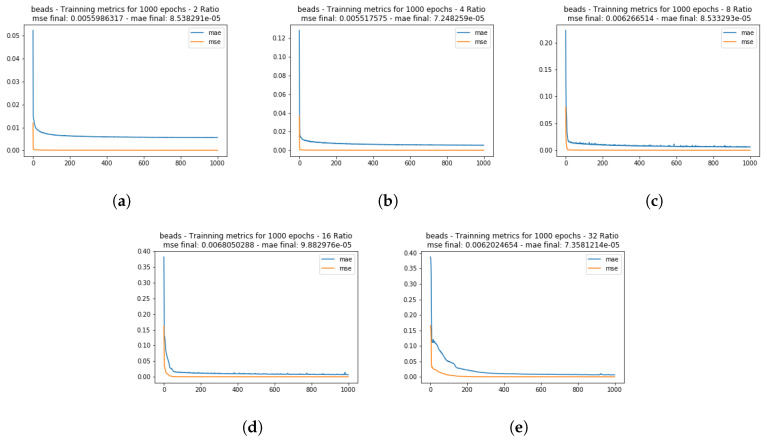
Mean squared error (MSE) and mean absolute error (MAE) training metrics for the CAVE dataset images, for the ratios of 2 (**a**), 4 (**b**), 8 (**c**), 16 (**d**), and 32 (**e**), with resolutions of 256 × 256, 128 × 128, 64 × 64, 32 × 32, and 16 × 16 pixels, respectively. Similar results were found for the other images tested.

To validate the training in each iteration for each set of images, the MSE, MAE, and $R^{2}$ were computed. The Figure 5 shows the MSE and MAE for different scale ratios in the CAVE dataset indicating a fast error minimization curve. Highlighting that, the sets with less data (when the input images were divided by 32) had a longer descent time to minimize the error metrics.

**Figure 6:**
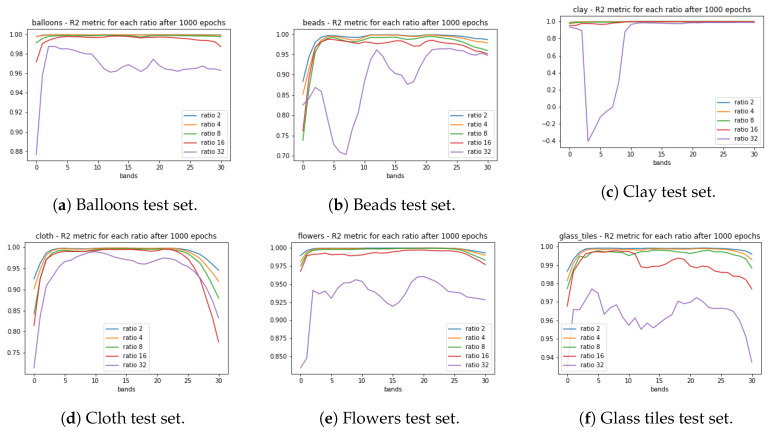
$R^{2}$ test set correlation metric for the CAVE dataset images chosen for the model validation. In each graph, the $R^{2}$ is computed for each of the 31 bands.

The Figure 6 shows the $R^{2}$ metric for each test set used during training for each set of images of the CAVE dataset. Again, as we have less data (ratio 32) the $R^{2}$ correlation became weaker, but still show a strong correlation between the desired and actual outputs, highlighting that some bands were better generated than others in all iterations, but, with $R^{2}$ values greater than 0.70.

With the models trained, the spectral images were generated aiming the original full resolution of 512 × 512 of the RGB original image as input. The generated spectral images and original images were compared against each other to generate the spectral image qualities SAM, UQI, RMSE, and ERGASS.

**Figure 7:**
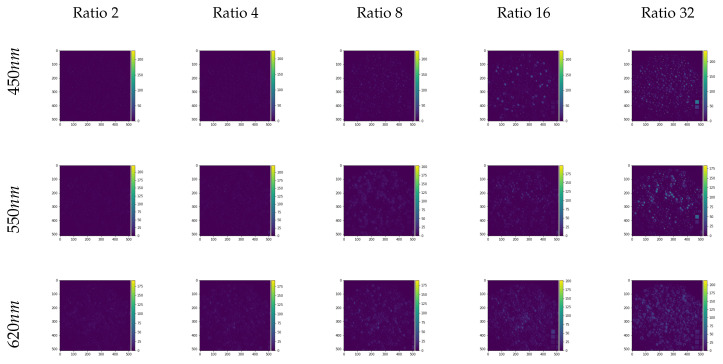
Image differences between the generated images and the original multispectral bands for the beads image on the CAVE dataset. The bands shown are equivalent to the work of [22].

Figure 7 shows scaled image differences in the selected bands in the wavelengths 450 nm, 550 nm, and 620 nm where the bright spots indicate greater differences between generated and original bands for the “beads” set as an example, showing great overall accuracy.

Table 1, Table 2, Table 3, Table 4 and Table 5 present the spectral quality index values for the set tested in this work, showing results close to the ideal, with these values naturally getting worse as we provide less data for the neural network (Table 4 and Table 5), but still presenting good results.

#### 3.1.2. Landsat Test Set

**Figure 8:**
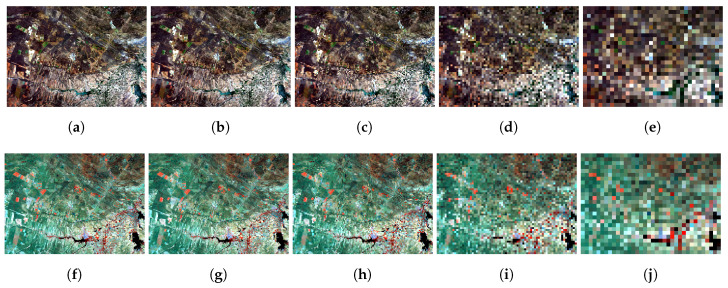
Landsat test set input for the ratios of 2 (**a**,**f**), 4 (**b**,**g**), 8 (**c**,**h**), 16 (**d**,**i**) and 32 (**e**,**j**), with resolutions of 581 × 419, 290 × 209, 145 × 104, 72 × 52, and 36 × 26 pixels.

Following the same parameters as the CAVE dataset tests, a larger area in the region of the main study was chosen to validate the methodology in a satellite image. For this test, the input set for each training iteration was configured as shown in Figure 8.

**Figure 9:**
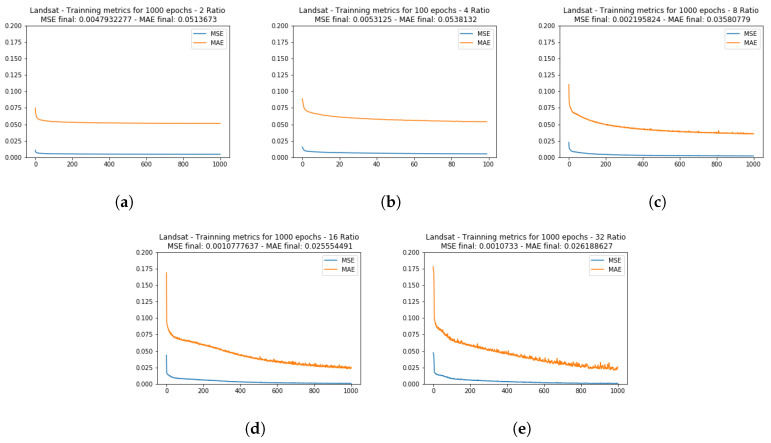
Mean squared error (MSE) and mean absolute error (MAE) training metrics for the Landsat dataset test image, for the ratios of 2 (**a**), 4 (**b**), 8 (**c**), 16 (**d**), and 32 (**e**).

**Figure 10:**
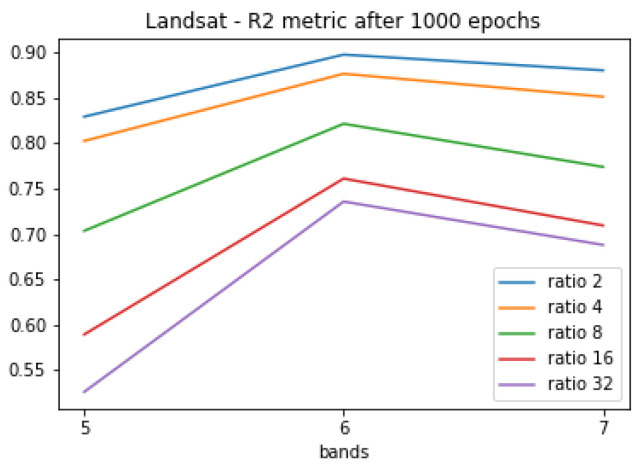
$R^{2}$ test set correlation metric for the Landsat test set.

The Figure 9 and Figure 10 show the learning metrics MSE and MAE for the Lansat test image set. As with the CAVE dataset, the learning curve in the tests with fewer data (ratio 32) presented a slower curve descent. For the $R^{2}$ metric, the near-infrared band (Landsat band 5) presented a lesser correlation compared with the other two bands.

**Figure 11:**
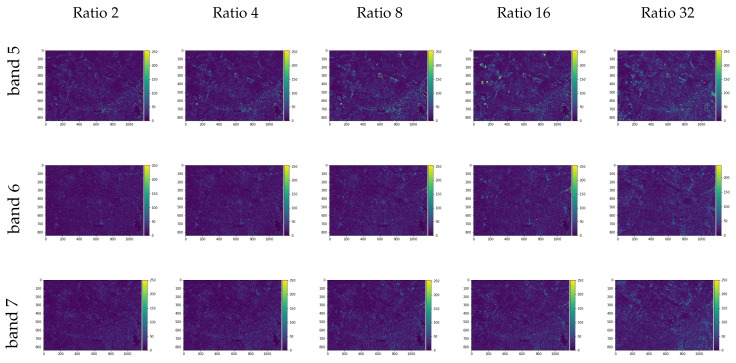
Image differences between the generated images and the original near-infrared (NIR) (band 5) and short-wave infrared (SWIR) (bands 6 and 7) for the Landsat image test set.

With the models trained, as defined in methodology, the original size Landsat input RGB composite was used to reconstruct the same size NIR and SWIR bands. The Figure 11 shows the image difference for each band and each trained model given the respective resized image ratios used as input training, where the pixels with brighter colors shows greater differences when comparing the original and the generated bands. The Table 6 presents the quality spectral indexes SAM, UQI, RMSE, and ERGASS given the original multispectral composition and the predicted composition of the spectral bands chosen.

#### 3.1.3. Increasing the Landsat Images with the Google Earth RGB Image

After testing the Super-Resolution method in the synthetic CAVE dataset and in the Landsat test set, we applied the same method for the final set for the studied area with images obtained from the Landsat 8 satellite (NIR and SWIR bands) and from the Google Earth RGB images (Digital Globe/GeoEye satellites) as input, varying from the Landsat test set that only used images/bands from the same satellite.

This step also differs from the prior training evaluations, by containing only one set with the RGB images with the same size as the Landsat 6 NIR and SWIR images. The quality evaluation is performed by analyzing the proper output image given the higher resolution RGB image used as input in the trained neural network using 2, 4, 6, and 16 times the width and height of the Landsat images.

To generate the higher spatial resolution NIR and SWIR bands, first an input set of 30 m spatial resolution was used, with the Google Earth RGB image degraded to this resolution.

**Figure 12:**
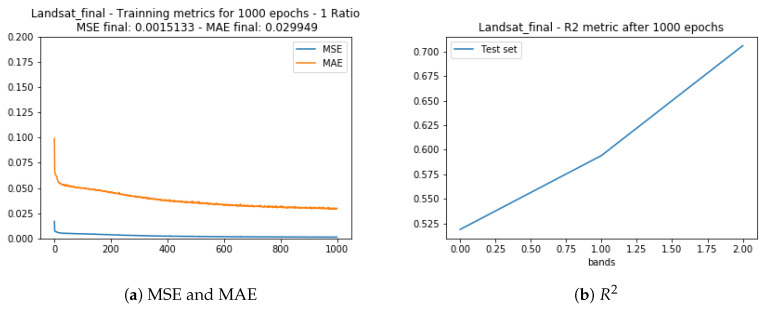
Training statistics for Landsat 8 and Google Earth images with 30 m spatial resolution.

The Figure 12 show the training evaluation metrics MSE, MAE, and $R^{2}$ for the test set obtained. As observed in the Landsat test set (prior subsection) the correlation between the generated and desired data was worse for the band 5 (NIR) and slightly better for the other two bands.

**Figure 13:**
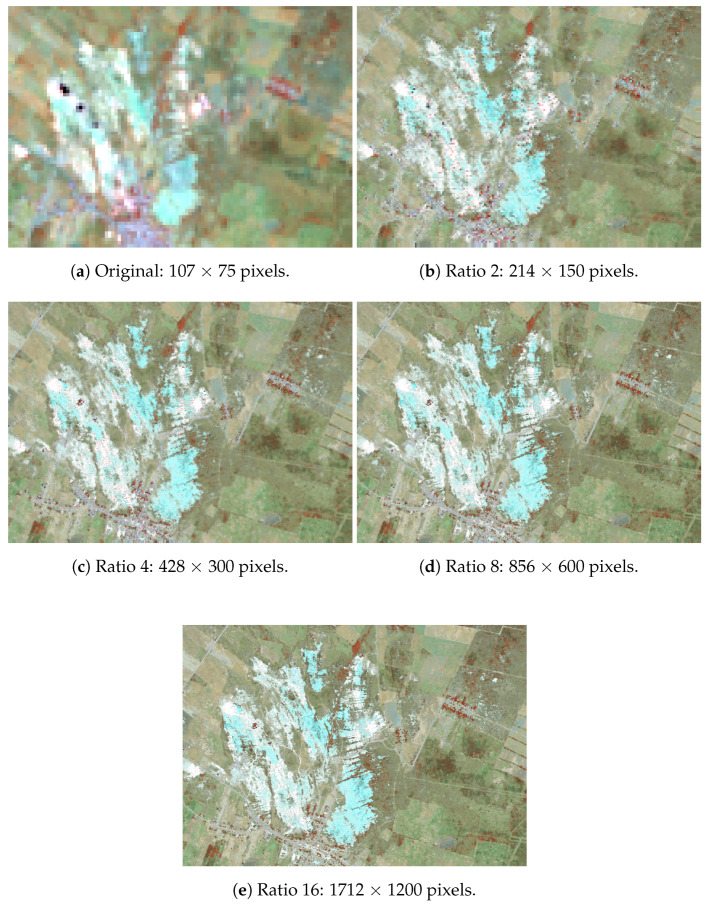
Generated images with different resolutions given the RGB input in a trained network with 30 m resolution images.

After the training step, we supplied to the network the different resolution RGB images to obtain higher spatial resolution NIR and SWIR bands. Figure 13 shows the colored composition of the original and generated products with a higher spatial resolution for visual comparison, where is noticeable an absent feature present in the original image probably by not represent enough data for training.

**Figure 14:**
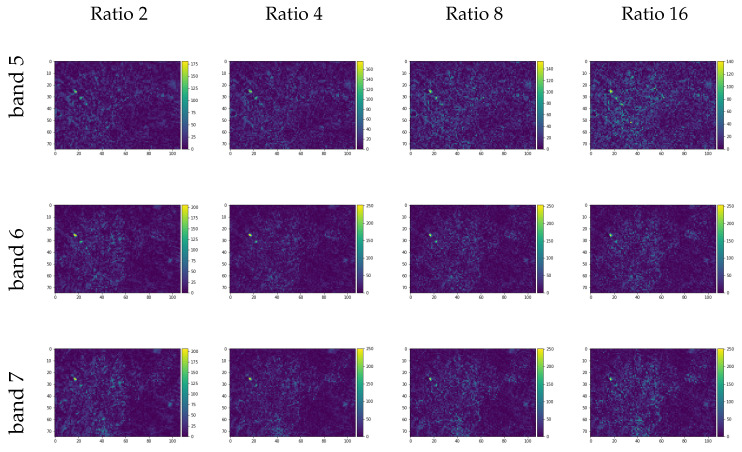
Image differences between the generated images and the original NIR (band 5) and SWIR (bands 6 and 7) for the Landsat set brought to 30 m pixel size following the Wald’s protocol.

These differences can be seen in Figure 14 where the brighter spots indicate the major differences between the generated and original bands brought to the same size, in this case with pixels of 30 m. Spectral quality indexes for this set are shown in the Table 7 with results close to the ideal for SAM and UQI indexes.

**Figure 15:**
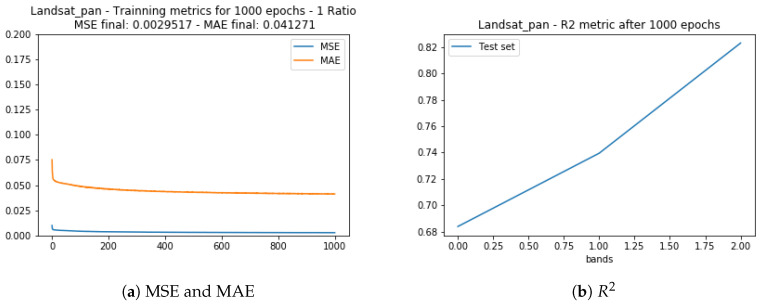
Training statistics for Landsat 8 and Google Earth images with 15m spatial resolution.

In order to obtain higher spatial resolution and recover more detail, a 15 m pixel resolution set was also used as training data for the same area. This set was obtained using the SCP plugin that allows pansharpening processing together with the satellite image download while the Google Earth RGB image was resized accordingly. The training validation statistics MSE, MAE, and $R^{2}$ test set correlation are shown in Figure 15, where the $R^{2}$ value is higher than the values obtained using the 30 m resolution training set.

**Figure 16:**
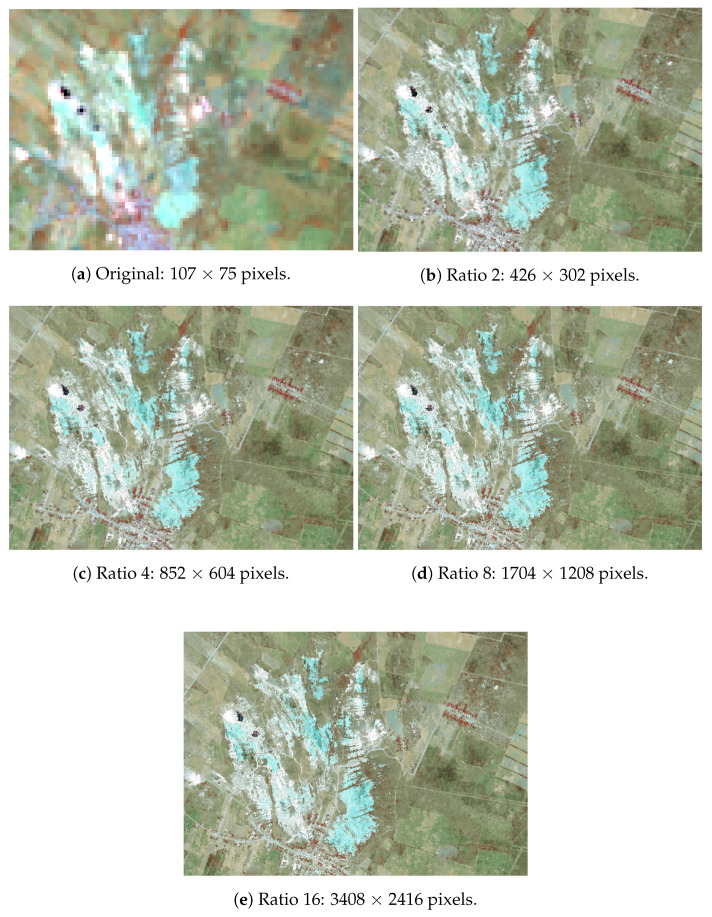
Generated spectral image compositions with different resolutions given the equivalent resized RGB image used as input in the trained neural network with 15 m pixel size images.

With the neural network trained, we generated the NIR and SWIR bands with higher spatial resolution supplying the RGB images to the network, each one with width and height resolution of the original Landsat band multiplied by 2, 4, 8, and 16. The NIR and SWIR (bands 5, 6, and 7) composites are shown in Figure 16 alongside the original multispectral composition. This set of images shows a water region detailed that was missing from the images shown in Figure 13.

**Figure 17:**
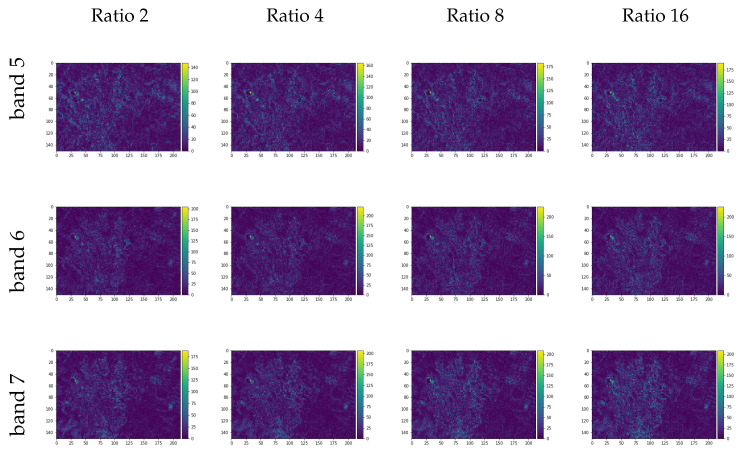
Image differences between the generated images and the original NIR (band 5) and SWIR (bands 6 and 7) for the Landsat final set brought to 30 m pixel size following the Wald’s protocol.

Image differences between the generated and original individual bands are presented in Figure 17 showing minor differences, corroborated by Table 8 spectral quality indexes values. As before, following the Wald’s protocol, the generated images were resized to the pansharpened Landsat images resolution.

Facing the satisfactory spatial resolution increasing using the method proposed we proceed with the image classification to better delineate the objects of interest using the composition with width and height multiplied by 16.

### 3.2. Classification

**Figure 18:**
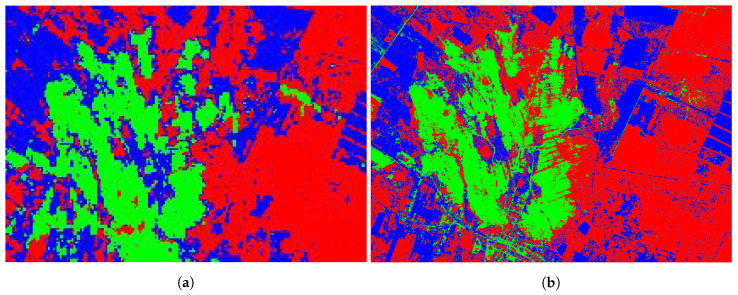
Classified RGB compositions. The areas in green are exposed soil (outcrop), the areas in red are grassland, and the areas in blue are dense vegetation or forest. (**a**) Classified Landsat 8 NIR and SWIR bands composition with 15m pixel resolution. (**b**) Classified predicted NIR and SWIR bands composition with pixel size of 1m approximately.

The classified images are presented in Figure 18b while the quantitative metrics are in Table 9. Considering the classified image is clear, the predicted high spatial resolution image shows more visible features that seem lost in the original image, although there is no ground truth for the generated image.

Analyzing the confusion matrix of the Landsat 8 image (Table 9), we can verify the high hit rating of the classifier. Of the 461 pixels selected randomly into the ROIs by ENVI software, 446 were classified correctly, achieving an accuracy of 96.74%, while the Precision and Recall reached more than 98% for the original image in Figure 18a.

The classification results for the increased spatial resolution Landsat 8 image is shown in Table 10. Despite the higher pixel count, the classifier identified the classes correctly relatively more times than in the original spatial resolution image.

## 4. Discussion

The spatial resolution problem has been explored with more intensity in the last years, and the advances in machine learning techniques and computational power have greatly contributed to the increased interest in generating multispectral and hyperspectral images with higher spatial resolution, especially due to the expensiveness of these kind of sensors to generate these images.

Prior works, in this regard, applied distinct techniques to increase the spatial resolution, varying from sparse data recovery, non-supervised clustering to deep convolution neural networks. To narrow down our scope we considered works that used RGB images as guides to obtain multispectral data like in [24,32,33,35,50,63].

Common to these works, controlled datasets offer a mean of comparison between the different techniques. The CAVE [45] and Harvard dataset [64] were the most present dataset complemented by the NUS dataset [24] and ICVL [26]. Other conference-related datasets are also widely used, although further access to these datasets can be difficult.

As we focused on RGB based spatial resolution increasing of multispectral data and the use of a common dataset for testing, the CAVE dataset was the chosen dataset. This dataset was also used for hyperspectral recovery, unmixing, and of course upsampling, and Super-Resolution in the works of [22,25,31,34,63]. Below, we will make some considerations between our results and the results found in these previous works.

The works of [22,25] used a ratio of 16 (32 × 32 pixels resolution) for the lower resolution images of the CAVE dataset, while the work of [31] used a ratio of 32 (16 × 16 pixels resolution) for the same set of images. Although, the works of [50,63] used the same dataset and evaluation methodology, the main objective was the spectral recovery instead of the resolution upsampling.

With the ratio of 16, ref. [25] obtained an average RMSE of 2.6 and a SAM of 6 while in this work we obtained a mean RMSE of 4.87 and a minimum of 2.5 and a maximum of 9.3, however not all the elements in the CAVE dataset were tested in our work. The SAM index for the images upscaled from this resolution resulted in values between 4 and 20 for the tested elements.

The work of [22], differently from [25] did an approach similar to our work showing the results for an individual set of images in the CAVE dataset. However, only two sets are the same, beads and “ballons”. According to the results shown in [22], they achieved for ballons and beads, RMSE values of 1.64 and 6.92, respectively. We achieved slightly higher RMSE values, obtaining 2.48, and 9.32 for balloons and beads sets respectively. Additionally, we achieved better results in some cases than [65,66], despite our more straightforward method.

Inspired by the NTIRE contest, advancements in computational power and neural network architecture like the convolution neural networks for Super-Resolution, newer works like [31,50,63], also performed evaluation tests using the same dataset used in our work.

The work of [31] also evaluated the upscaled images with RMSE, SAM, and ERGAS metrics. The method named Partial Dense Connected Spatial and Spectral Fusion (PDCon-SSF) obtained 2.18, 4.38, and 0.22 for the RMSE, SAM, and ERGAS, respectively. Compared with this work we achieved much higher values of RMSE and SAM, but with lower values for ERGAS in general.

Wrapping up, we can observe that our method presented equal or better results than previous work employing a simpler method than the CNNs and the composite methods already employed in some works with the advantage of requiring less training time, from minutes to half-hour for the images in the CAVE dataset instead of more than 30 h as seen in the works presented in [36]. However, each network trained is specialized to a given image.

Considering the Landsat area used for the method evaluation, our results were near to the ideal in all scales, however, no similar test routines were identified for Landsat data (training with downscale images and validating with the original images). Recent similar works, like [19,67], increased the spatial resolution of satellite images, but with some caveats. The work of [19] inspired by the work of [30] achieved good results compared with parametric pansharpening methods when increasing the resolution of Geo-Eye, Ikonos, and WorldView 2 satellite images. The work of [67] increased spatial resolution of Landsat satellite images using CNN also applying a temporal factor, but training with Sentinel-2 images.

Finally, considering our final study case, training with downsized high-resolution RGB images obtained from Google Earth, minimal spectral distortions were found with values near the ideal for the quality indexes evaluated. However, two concerns deserve more attention: the first is that the Google Earth service provide a collated image from satellites that have distinct characteristics, and also, the acquisition date can vary greatly. As a result of this, we had to avoid use a larger area visibly composed of different satellites, which could make the NN not to learn the desired patterns; the second one is related to the number of pixels representing each identifiable feature. We could observe this comparing the Figure 13 and Figure 16. The generated higher resolution compositions in Figure 13 lack the water region (in black) that is present in the original image. This is not an issue in Figure 16, where much more pixels (due to the double input resolution) are present during the learning stage to represent this object.

As there is no major difference between the different up-scaled resolutions, we choose the image composition with the highest spatial resolution (with pixel size close to 1 m) to perform the spectral classification.

It is important to point out that the random choice of pixels is made based on the number of regions of interest selected in the classification process. Due to the differences in the number and size of polygons (ROIs), some classes have more analyzed pixels than others.

From 106 pixels selected by the algorithm as Grassland on the original image, 10 pixels were classified as Forest. This misclassification can be associated with the spectral similarity of the materials (Grassland and Forest), while 3 pixels were classified as Exposed Soil (outcrop). It is noteworthy that the Exposed Soil was the only class with no misclassified pixels, all the 107 pixels were correctly classified by the Maximum Likelihood method. The kappa index showed an almost perfect agreement between the classified data and the ground truth data, reaching a value of 0.9456. The MCC reached was 0.9626, close to a completely correct classification (MCC=1).

The confusion matrix of the Super-Resolution classified image showed a high hit rate. Due to the higher spatial resolution, the algorithm selected a higher number of pixels into the ROIs, which provided a bigger dataset to validate the classification.

The algorithm used 448,554 pixels to validate the classification and 436,146 pixels were classified correctly. Despite more than 12,000 pixels were incorrectly classified, the pixels correctly classified represented an accuracy of 97.23%. Considering that the pixel count is greater than in the original Landsat 8 image, the overall accuracy obtained from the Super-Resolution image classification is greater than the Landsat 8 image classification with the difference between the accuracies lower than 0.1. The precision and recall reached more than 98%. The difference in the values of precision shows that the classification of the predicted image generated a lower false-positive rate so that the lower value of the recall shows a lower false-positive rate for the predicted image.

From the validation process performed with the Landsat 15 m dataset, it is possible to verify some confusions committed by the classifier. All the classes had misclassified pixels, the Forest is the class with the highest number of incorrectly classified pixels. Of the 218,749 pixels assigned as Forest in the ground truth dataset, 89 pixels were classified as Exposed Soil and 6560 pixels were classified as Grassland. As the Landsat 8 classification, there is still a confusion between the Grassland and the Forest. The Exposed Soil is still the class with the lower rate of misclassified pixels, of the 36,097 pixels, 1647 were classified as Grassland, and 385 were classified as Forest. The kappa index reached was 0.9513, higher than the index for Landsat 8 classification, representing an almost perfect agreement. The MCC reached was 0.9716, very close to 1, higher than the MCC for Landsat 8 image classification.

## 5. Conclusions

This work presented a method to increase the spatial resolution of multispectral images with a single RGB image from Google Earth and an ANN, to better outline exposed land soil or outcrops which are of the great interest for petroleum industry.

The methodology considers neighborhood pixels in kernels as a strategy to provide more data to stretch the spectrum by estimating new spectral channels for the limited dataset available in high spatial resolution images. To assess the proposed method, images were simulated by gradually decreasing the scale of the image and application of the proposed method to return the original configuration, while the original image was kept for reference. The quality evaluation showed results close to the ideal with minimum spectral distortion as seen in the result section,

The method in which image quality tests for both synthetic datasets where applicable showed similar results to the related works, demonstrating the validity of the proposed methodology as an alternative to the trend of CNN variations for spectral reconstruction without relying on large datasets for training.

The experiments showed some inverse proportionality between the Super-Resolution upscaling and the quality indexes, higher spatial resolution upscaling offers worse metrics overall. This result was expected, and although we do not draw a rigid limitation for the proposed technique, we offered evidence on the quality expected on spatial resolution improvements of ratios 2, 4, 8, 16, and 32.

In this work, the trade-off whereabouts concerning the difference between the input and output spatial resolutions was established. Moreover, the experiments strongly indicated that there is a remarkable relationship between the characteristics of the targets in the image and the ability of the method to retrieve reliable results.

In a real-world experiment, the method was applied to an area of interest for petroleum geology including carbonate outcrops. The high-resolution image could improve the delineation of the study area providing improved input data for a pre-field evaluation.

Future works include the exploration of new satellite datasets and the use of carbonate indices to identify the areas of interest, as well as the improvement of the methodology as a function of characteristics of input data like size and shape of targets in the image.

## Figures and Tables

**Table 1:** Spectral quality indexes for the higher spatial resolution CAVE images trained with input of 256 × 256 pixels.

Ratio 2	Balloons	Beads	Clay	Cloth	Flowers	Glass Tiles	Lowest	Highest	Mean
**SAM**	0.019	0.081	0.042	0.055	0.053	0.054	0.019	0.081	0.051
**RMSE**	1.099	3.859	1.091	2.880	2.180	2.660	1.091	3.859	2.295
**ERGAS**	1.106	4.687	3.125	2.774	3.966	3.265	1.106	4.687	3.154
**UQI**	0.967	0.971	0.795	0.995	0.849	0.980	0.795	0.995	0.926

**Table 2:** Spectral quality indexes for the higher spatial resolution CAVE images trained with input of 128 × 128 pixels.

Ratio 4	Balloons	Beads	Clay	Cloth	Flowers	Glass Tiles	Lowest	Highest	Mean
**SAM**	0.026	0.099	0.053	0.060	0.056	0.073	0.026	0.099	0.061
**RMSE**	1.423	4.641	1.573	3.292	2.415	3.594	1.423	4.641	2.823
**ERGAS**	0.357	1.439	0.983	0.782	1.079	1.102	0.357	1.439	0.957
**UQI**	0.970	0.973	0.795	0.995	0.858	0.981	0.795	0.995	0.929

**Table 3:** Spectral quality indexes for the higher spatial resolution CAVE images trained with input of 64 × 64 pixels.

Ratio 8	Balloons	Beads	Clay	Cloth	Flowers	Glass Tiles	Lowest	Highest	Mean
**SAM**	0.033	0.112	0.063	0.067	0.059	0.094	0.033	0.112	0.071
**RMSE**	1.929	5.721	1.815	4.020	2.701	4.663	1.815	5.721	3.475
**ERGAS**	0.121	0.429	0.289	0.235	0.292	0.363	0.121	0.429	0.288
**UQI**	0.969	0.968	0.811	0.992	0.857	0.982	0.811	0.992	0.930

**Table 4:** Spectral quality indexes for the higher spatial resolution CAVE images trained with input of 32 × 32 pixels.

Ratio 16	Balloons	Beads	Clay	Cloth	Flowers	Glass Tiles	Lowest	Highest	Mean
**SAM**	0.046	0.201	0.201	0.089	0.073	0.126	0.046	0.201	0.123
**RMSE**	2.481	9.322	4.859	3.308	3.308	5.977	2.481	9.322	4.876
**ERGAS**	0.039	0.197	0.269	0.069	0.089	0.118	0.039	0.269	0.130
**UQI**	0.974	0.941	0.775	0.991	0.829	0.980	0.775	0.991	0.915

**Table 5:** Spectral quality indexes for the higher spatial resolution CAVE images trained with input of 16 × 16 pixels.

Ratio 32	Balloons	Beads	Clay	Cloth	Flowers	Glass Tiles	Lowest	Highest	Mean
**SAM**	0.121	0.220	0.413	0.124	0.230	0.179	0.121	0.413	0.215
**RMSE**	6.383	11.118	8.521	5.464	9.848	8.444	5.464	11.118	8.296
**ERGAS**	0.025	0.055	0.120	0.023	0.077	0.042	0.023	0.120	0.057
**UQI**	0.947	0.921	0.608	0.983	0.781	0.920	0.608	0.983	0.860

**Table 6:** Spectral quality indexes applied to Landsat test set with training inputs of 581 × 419 (Ratio 2), 290 × 209 (Ratio 4), 145 × 104 (Ratio 8), 72 × 52 (Ratio 16), and 36 × 26 pixels (Ratio 32).

Ratio	SAM	RMSE	ERGAS	UQI
**2**	0.171	21.770	6.797	0.964
**4**	0.187	23.777	1.847	0.959
**8**	0.217	27.590	0.532	0.948
**16**	0.240	30.247	0.145	0.941
**32**	0.241	31.240	0.038	0.933

**Table 7:** Landsat spectral quality indexes between the generated and original spectral compositions resized to 30 m pixel resolution.

Multiplier	SAM	RMSE	ERGAS	UQI
**2**	0.110	17.830	16.022	0.993
**4**	0.113	18.294	16.443	0.993
**8**	0.127	20.618	18.551	0.991
**16**	0.141	22.955	20.658	0.989
**32**	0.153	24.869	22.381	0.987

**Table 8:** Landsat spectral quality indexes between the generated and original spectral compositions resized to 15 m pixel resolution.

Multiplier	SAM	RMSE	ERGAS	UQI
**2**	0.112	17.795	16.469	0.992
**4**	0.123	19.421	17.996	0.991
**8**	0.139	21.924	20.330	0.989
**16**	0.150	23.655	21.940	0.987

**Table 9:** Landsat 8 image classification confusion matrix and evaluation measures. The percentage values are related to the number of classified pixels in the image.

Class	Grassland	Forest	Exposed Soil	Total
Grassland	93 (97.89%)	10 (3.91%)	03 (2.73%)	106 (22.99%)
Forest	02 (2.11%)	246 (96.09%)	00 (0%)	248 (53.8%)
Exposed Soil	00 (0%)	00 (0%)	107 (97.27%)	107 (23.21%)
Total	95 (100%)	256 (100%)	110 (100%)	461 (100%)
Accuracy		0.9674		
Precision		0.9802		
Recall		0.9824		
Kappa		0.9456		
MCC		0.9626		

**Table 10:** Predicted image classification confusion matrix and evaluation measures. The percentage values are related to the number of classified pixels in the image.

Class	Grassland	Forest	Exposed Soil	Total
Grassland	189,981 (95.86%)	3726 (1.72%)	01 (00%)	193,708 (43.18%)
Forest	6560 (3.31%)	212,100 (98.10%)	89 (0.26%)	218,749 (48. 77%))
Exposed Soil	1647 (0.83%)	385 (0.18%)	34,065 (99.74%)	36,097 (8.05%)
Total	198,118 (100%)	216,211 (100%)	34,155 (100%)	448,554 (100%)
Accuracy		0.97		
Precision		0.98		
Recall		0.98		
Kappa		0.95		
MCC		0.97		
